# Optimizing PMMA solutions to suppress contamination in the transfer of CVD graphene for batch production

**DOI:** 10.3762/bjnano.13.70

**Published:** 2022-08-18

**Authors:** Chun-Da Liao, Andrea Capasso, Tiago Queirós, Telma Domingues, Fatima Cerqueira, Nicoleta Nicoara, Jérôme Borme, Paulo Freitas, Pedro Alpuim

**Affiliations:** 1 International Iberian Nanotechnology Laboratory, Braga 4715-330, Portugal; 2 Centro de Física das Universidades do Minho e Porto (CF-UM-UP), Universidade do Minho, Braga 4710-057, Portugal

**Keywords:** 2D materials, graphene transfer process, large-scale fabrication, microelectronics, poly(methyl methacrylate)

## Abstract

Mass production and commercial adoption of graphene-based devices are held back by a few crucial technical challenges related to quality control. In the case of graphene produced by chemical vapor deposition, the transfer process represents a delicate step that can compromise device performance and reliability, thus hindering industrial production. In this context, the impact of poly(methyl methacrylate) (PMMA), the most common support material for transferring graphene from the Cu substrate to any target surface, can be decisive in obtaining reproducible sample batches. Although effective in mechanically supporting graphene during the transfer, PMMA solutions needs to be efficiently designed, deposited, and post-treated to serve their purpose while minimizing potential contaminations. Here, we prepared and tested PMMA solutions with different average molecular weight (AMW) and weight concentration in anisole, to be deposited by spin coating. Optical microscopy and Raman spectroscopy showed that the amount of PMMA residues on transferred graphene is proportional to the AMW and concentration in the solvent. At the same time, the mechanical strength of the PMMA layer is proportional to the AMW. These tests served to design an optimized PMMA solution made of a mixture of 550,000 (550k) and 15,000 (15k) AMW PMMA in anisole at 3% concentration. In this design, PMMA-550k provided suitable mechanical strength against breakage during the transfer cycles, while PMMA-15k promoted depolymerization, which allowed for a complete removal of PMMA residues without the need for any post-treatment. An XPS analysis confirmed the cleanness of the optimized process. We validated the impact of the optimized PMMA solution on the mass fabrication of arrays of electrolyte-gated graphene field-effect transistors operating as biosensors. On average, the transistor channel resistance decreased from 1860 to 690 Ω when using the optimized PMMA. Even more importantly, the vast majority of these resistance values are distributed within a narrow range (only ca. 300 Ω wide), in evident contrast with the scattered values obtained in non-optimized devices (about 30% of which showed values above 1 MΩ). These results prove that the optimized PMMA solution unlock the production of reproducible electronic devices at the batch scale, which is the key to industrial production.

## Introduction

Graphene and two-dimensional (2D) transition metal dichalcogenides (TMDCs) have been the focus of an intense research effort aimed at developing a new class of innovative devices and applications [1–3]. Among the production methods, chemical vapor deposition (CVD) made substantial progress over the years and now guarantees high-quality standards for the growth of batches of graphene samples over wafer-scale areas [4–6]. This progress allowed for the fabrication of a wide range of 2D material-based devices and heterostructures, especially in optoelectronics [7–9]. At present, one of the remaining challenges in the fabrication of graphene-based devices lies in the reproducibility: More than the CVD itself, the transfer process from the growth substrate (e.g., Cu or Ni) to the desired target substrate (e.g., SiO_2_/Si, glass, or flexible polymers) often introduces inconsistencies among devices [10]. Various approaches have been developed to address this issue and establish a reproducible transfer process [11–17]. Among the many, the poly(methyl methacrylate) (PMMA)-assisted process remains the most reliable and most commonly used approach [18]. The chemical structure of PMMA features long polymer chains, whose length is proportional to the average molecular weight (AMW) of the polymer. During the transfer of graphene, the polymer serves as a supporting layer to (i) retain the integrity of graphene during the wet-etching bath required to dissolve the metallic substrate and (ii) provide mechanical stability when transferring graphene to the target substrates. During this process, two primary external sources of contamination need to be considered: (i) metallic particles from the Cu or Ni etching process and (ii) PMMA residues after the removal and rinsing processes. Both contaminations are leading causes of undesired p-type doping in CVD graphene, accompanied by a deterioration of its electrical properties [19–22]. The metallic contamination from etchants such as FeCl_3_ can be substantially reduced by rinsing PMMA-coated graphene in DI water solution with 1–2% HCl [12]. Concerning the PMMA residues, several approaches were implemented to dissolve them, primarily by disrupting their chemical bonds. The chemical bond breakage is crucial, considering that PMMA solutions with higher weight percentage (wt %) are usually preferred as they form thicker supporting layers by spin coating: Such layers are mechanically more robust, yet leave behind significant residues [20]. Annealing processes (usually 200–450 °C, under an inert atmosphere or vacuum) were proposed [20], enabling depolymerization by breaking the molecular backbone bonds of PMMA [19,22–23]. Similarly, UV radiation can break the ester groups of PMMA, thus weakening the intermolecular interactions with graphene [24]. PMMA with higher AMW is harder to depolymerize due to strong van der Waals and London attractive forces among the long polymer chains [13]. It must also be considered that thermal treatments can often be counterproductive as they intensify polymerization, harden the PMMA residues, and complicate the removal.

Therefore, the way toward clean graphene processing appears to lie in the optimization of the PMMA-assisted transfer. In this context, we propose an optimized approach for a clean mass transfer of graphene samples over wafer-scale areas. A PMMA mixture was developed by balancing the AMW and weight percentage in anisole to guarantee a reliable transfer at a negligible contamination level, even without any post-treatment at high temperature. The supporting layer formed by spin coating presents high mechanical flexibility and strength for the transfer process and appears easy to dissolve afterward. We validated the impact of the optimized process in the mass fabrication of arrays of receded-gate graphene field-effect transistors for biosensing applications.

## Results and Discussion

**Figure 1:**
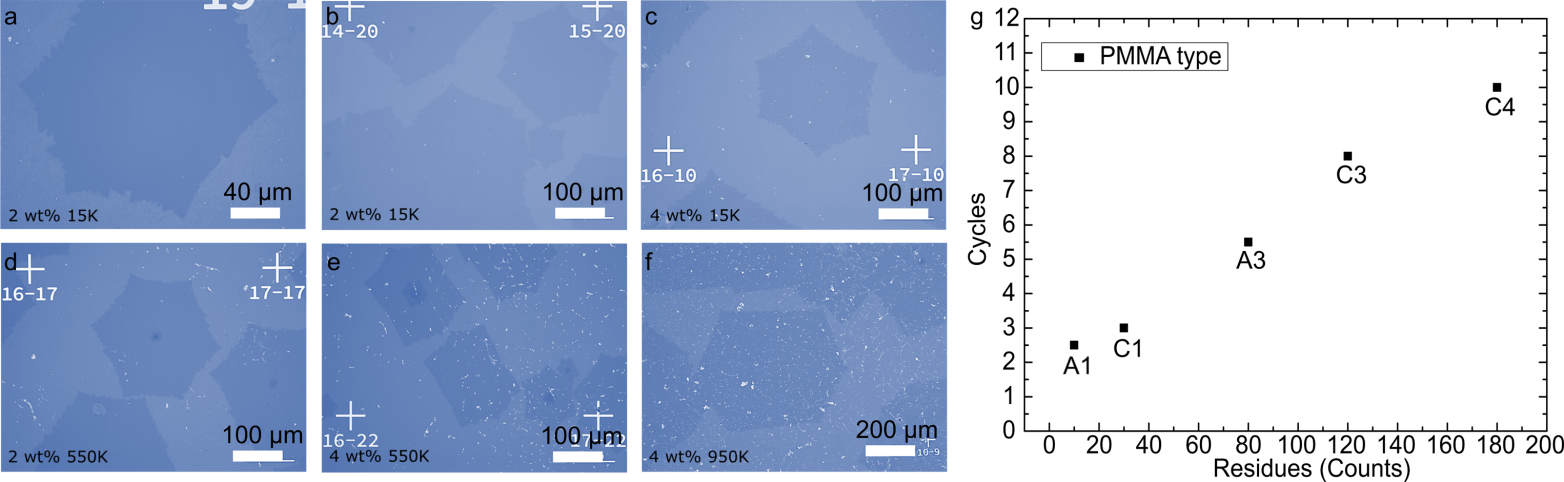
Examination of PMMA residues after the transfer of graphene single crystals using PMMA at various weight percentages and AMWs. (a, b) A1, (c) C1, (d) A3, (e) C3, and (f) C4. The white spots in the images are PMMA residues. The cross markers with coordinates are imprinted on the wafer to locate the graphene crystals. (g) Number of residue particles counted as function of the average molecular weight of PMMA.

We transferred graphene by using PMMA with two AMWs (15k and 550k), which were dissolved in anisole at two weight ratios (2 and 4 wt %). PMMA with 950,000 (950k) AMW (at 4 wt %), commonly used for microfabrication processes as an e-beam resist, was used for further comparison (see Experimental section, “Graphene transfer“). Optical microscopy analysis was carried out to visually evaluate the presence of PMMA residues after the transfer process of graphene single crystals using PMMA with different weight percentages and AMWs (Figure 1a–g). As detailed in the description of the graphene transfer process, after the Cu etching process (Supporting Information File 1, Figure S1b, step II), the PMMA-coated graphene is rinsed in a DI water bath at least three times (Supporting Information File 1, Figure S1b, step III). Each cycle includes two actions, namely (i) scooping up the sample and (ii) releasing it into the water bath. After the rinse process, the sample must be moved to a target substrate, which takes one more transfer cycle. Therefore, the wet transfer process entails at least four cycles. A sufficiently high mechanical strength of the supporting PMMA layer is the key requirement for a successful transfer. Figure 1g shows our experimental observations on the cleanness level and maximum number of transfer cycles afforded by each PMMA solution. For statistical purposes, the number of cycles for each test was extended to eight by transferring the PMMA/graphene between two aqueous solutions. A1 and C1 PMMA solutions (see Table 1 below for the sample denominations) allowed for less than four cycles without breakage, demonstrating a low mechanical strength. The strength appears to reach a proper level for A3 PMMA, which allowed for up to six complete cycles. C3 and C4 PMMA provided the highest mechanical support, allowing for up to ten cycles. To evaluate the process cleanness, the residues were quantified by counting the white spots (larger than 2.5 µm) in the images. Micrographs taken on 650 × 500 µm^2^ areas were compared (Figure 1a–f). The amount of PMMA residues appears to be directly related to the PMMA concentration. A1 showed very little residues (they can be almost completely removed using acetone) and low mechanical strength, whereas C1 provided proper support while maintaining a low residue level. The length of the PMMA molecular chains (proportional to molecular weight and the attractive intermolecular force) appeared to be the determining factor in the mechanical strength (as demonstrated by C3 PMMA, enabling eight complete transfer cycles). Overall, all 550k samples provided strong mechanical support yet translated into a moderate-to-high residue density level. C4 revealed the densest residue distribution due to extended molecular chains and the highest mass concentration.

**Figure 2:**
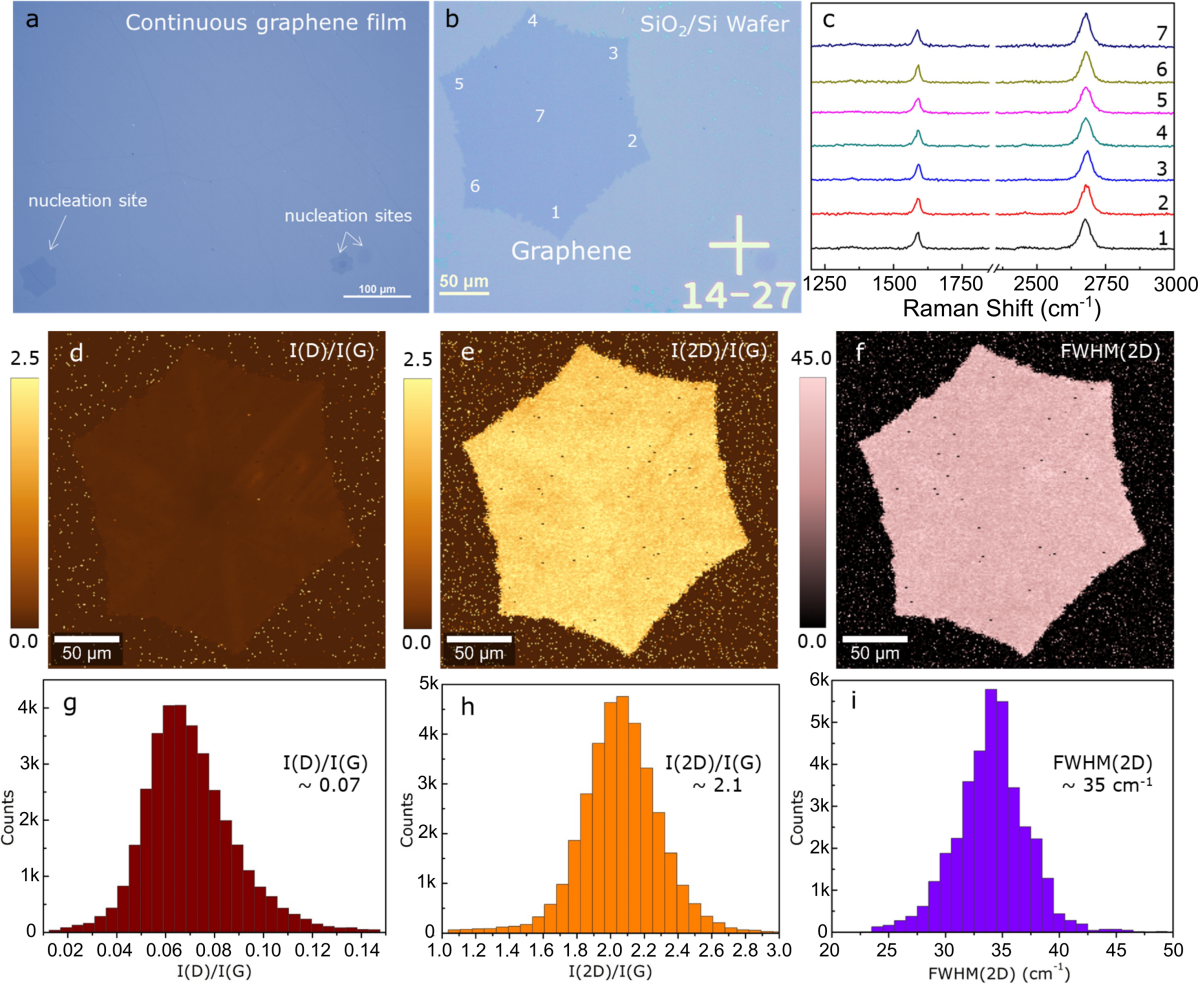
Raman analysis of graphene samples transferred with the optimized B2 PMMA solution. Optical images of (a) a large-area film and (b) a single crystal. (c) Raman spectra taken at the positions indicated in (b). Raman mapping of (d) *I*(D)/*I*(G), (e) *I*(2D)/*I*(G), and (f) FWHM(2D), and (g–i) corresponding statistics.

A PMMA mixture (coded B2, 3% mixture of PMMA-15k and PMMA-550k, see Table 1) was designed to lower the potential residue concentration while maintaining proper mechanical support. The rationale for the design of the optimized mixture is based on two hypotheses: (i) The PMMA-15k component provides short polymer chains, which are expected to diminish the molecular chain entanglement and, hence, the residue level. (ii) The sole presence of short polymer chains should, however, weaken the mechanical strength of the spin-coated layer. Therefore, the addition of PMMA-550k compensates for that and grants support during the transfer. B2 was tested in the transfer of both a graphene film and a single crystal (Figure 2a,b). The area analysis (650 × 500 µm^2^) revealed less than ten residues, indicating an extremely clean transfer process. B2 PMMA allowed for up to six transfer cycles, representing an intermediate, yet acceptable, mechanical support. This proves that the PMMA mixture features good mechanical strength and cleanness (i.e., the acetone bath can thoroughly remove it). The transferred graphene samples were investigated via Raman spectroscopy to evaluate crystallinity, layer number, and structural defect level [23]. The relative intensities of the G (ca. 1585 cm^−1^) and 2D (ca. 2700 cm^−1^) bands are typical of monolayer graphene [23–26]. The defect density appears minimal considering the negligible D band intensity at ca. 1350 cm^−1^ (Figure 2c) [27]. The Raman mapping in Figure 2d–i examines the whole crystal area [28]. The map and the corresponding statistics in Figure 2d and Figure 2g, respectively, show that the *I*(D)/*I*(G) ratio is very low (down to 0.03), meaning that no or few defects could be detected. Figure 2e and Figure 2h show that over 95% of the sample has a ratio of *I*(2D)/*I*(G) > 1.6 (average of 2.1 ± 0.3) and a FWHM(2D) of 34.2 ± 3.0 cm^−1^ (Figure 2f,i), implying high-quality monolayer graphene. Together, the data further support uniformity and crystallinity of the sample.

**Figure 3:**
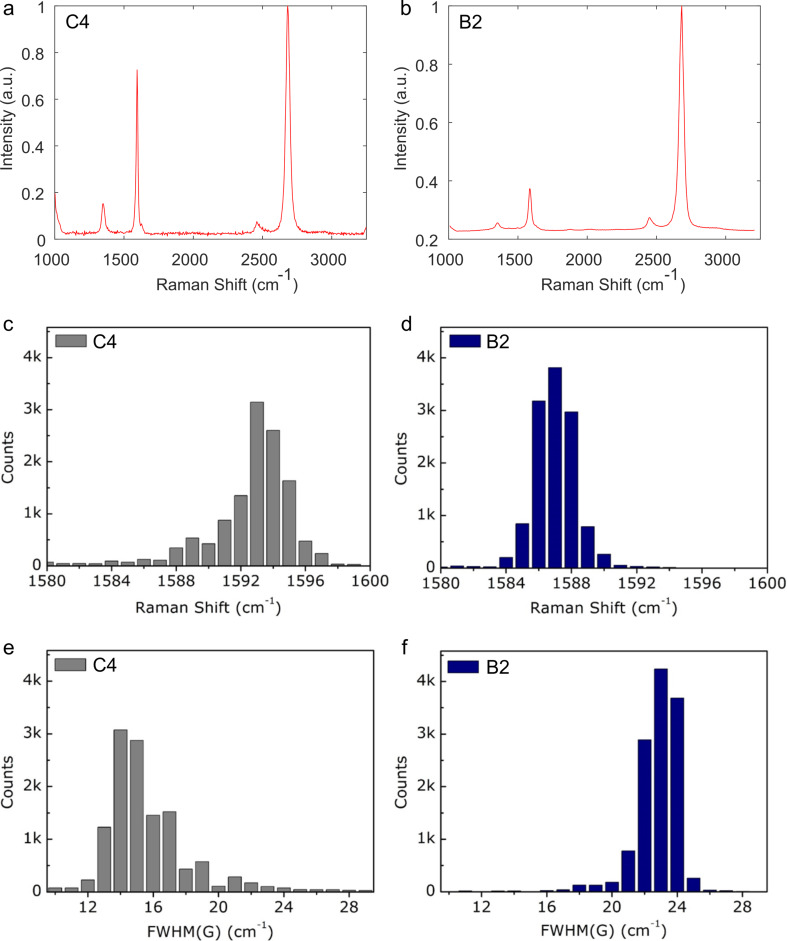
Statistical analysis of the Raman spectra of transistors prepared with transfers using PMMA mixtures with different molecular weights. (a, b) Representative Raman spectra of graphene transistor channels prepared using B2 and C4 mixtures, respectively. (c, d) Distributions of the G band peak position for B2 and C4, respectively. (e, f) Distributions of the FWHM of the G band.

The G phonon band arises from double degeneracy of iTO and iLO phonon modes (*E*_2_*_g_* symmetry) at the Brillouin zone center, which is an in-plane vibration of sp^2^ carbon atoms [23], and its position displays a blueshift as the charge carrier concentration rises. That is, the frequency shift of the G band is proportional to |*E*_F_|, which sets the carrier concentration. Due to the method and materials employed for the graphene transfer being the same except for the PMMA mixture, we consider that the differences in Raman spectra between the different samples can be attributed to adsorbed PMMA residues. Such residues could absorb water and oxygen molecules, conferring p-type doping to graphene [29]. Conversely, by tracking the G phonon band features in Raman spectra, we can correlate the degree of doping in graphene [30–31] with PMMA residues. A comparison of the statistical data of the G band position and FWHM(G) of graphene transferred with C4 and B2 PMMA is presented in Figure 3. Representative Raman spectra for the two cases are shown in Figure 3a and Figure 3b, respectively. The comparison of Figure 3c and Figure 3d shows that the G peak position is blueshifted in C4 samples compared to B2 samples (from an average of 1587 to 1593 cm^−1^), indicating that the charge carrier concentration did not rise as much when using B2 PMMA. This shows that B2 leaves behind a much lower (if any) density of residues. This redshift observed upon reducing the PMMA residue concentration is consistent with studies on advanced methods for cleaning PMMA from graphene [32]. The average FWHM(G) for the C4 and B2 samples is 14 and 23 cm^−1^, respectively (Figure 3e and Figure 3f). The broader G phonon band observed for the B2 samples reveals that a significantly higher number of inter-band decay pathways are available due to a lower Pauli blocking threshold [23] (equivalent to twice |E_F_|), further indicating that the p-type doping caused by adsorbed PMMA is less intense for the B2 samples. This result again supports that employing B2 PMMA yields fewer residues and may help in avoiding post-transfer treatments for advanced PMMA residue cleaning of graphene, such as annealing and ion beam irradiation [32]. The graph of the G band shift (Supporting Information File 1, Figure S2a) confirms that C4 PMMA leaves the highest level of contamination. This case also shows the highest standard deviation of the G band shift and FWHM(G) (Supporting Information File 1, Figure S2a,b) due to heterogeneous doping levels in the sample. We explain these results by the higher variance in the proportions of PMMA residue aggregates, resulting in alternating regions of intensively local p-type doping (large PMMA aggregates) and regions of less intense p-doping (small PMMA aggregates). In contrast, PMMA mixtures with lighter AMWs showed lighter and more uniform p-type doping over the crystallite area (smaller error bars). Few-layer crystals transferred with B2 are analyzed in Supporting Information File 1, Figure S3. The graphene crystal in Supporting Information File 1, Figure S3a,b is composed of four layers having a thickness of 0.4–0.5 nm (Supporting Information File 1, Figure S3c) [28,33]. The crystal morphology appears very smooth and free of identifiable impurities, with an average surface roughness (*R*_a_) of ca. 0.2 nm (Supporting Information File 1, Figure S3d). This value is one order of magnitude lower than that of graphene crystals transferred with C4 PMMA, which showed a surface roughness of ca. 2.8 nm. The low roughness obtained with B2 PMMA can be also related to a minimal occurrence of nanometer-scale PMMA residues.

**Figure 4:**
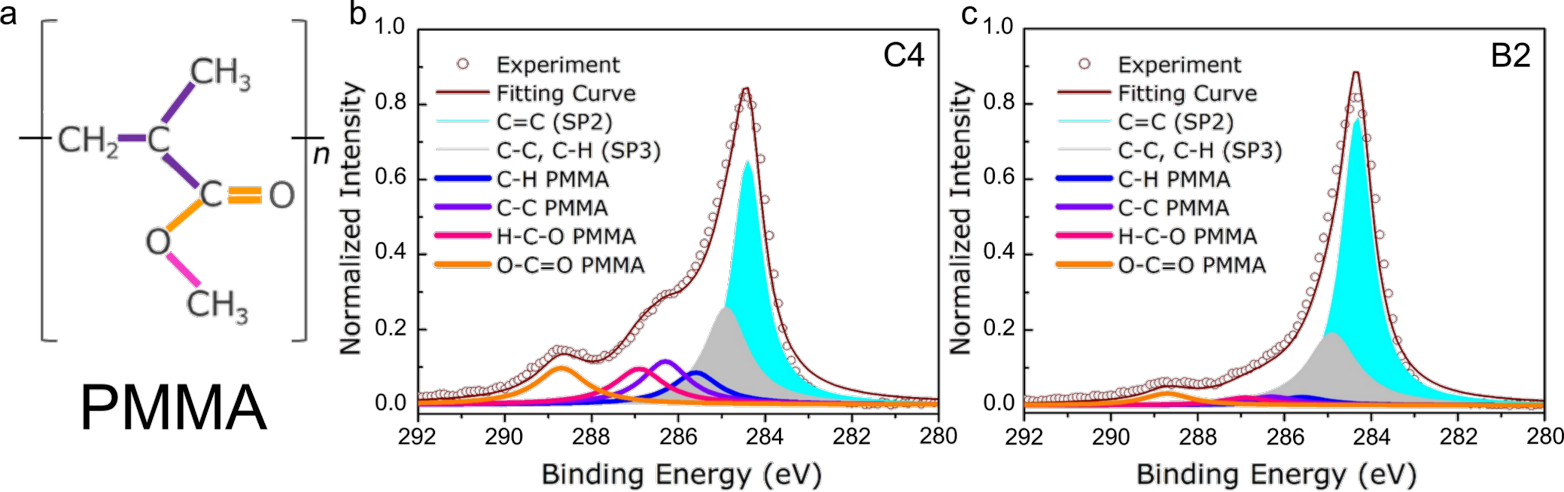
(a) Representation of the molecular structure of PMMA. XPS C1s spectra of graphene samples transferred using (b) C4 and (c) B2. The normalized spectra are fitted by Gaussian–Lorentzian curves. The solid blue and grey fills identify, respectively, sp^2^- and sp^3^-hybridized carbon bonds in graphene, located at ca. 284.4 and 285.0 eV, respectively. C–H (blue line), C–C (purple line), O–CH_3_ (pink line), and O–C=O (orange line) bonds are located at ca. 285.7, 286.3, 287.0 and 289.0 eV, respectively.

XPS was employed to analyze the graphene samples transferred using C4 and B2 PMMA (Figure 4). Figure 4a shows the chemical structure of the PMMA molecule. The C1s spectra can be decomposed into two prominent peaks originating from sp^2^-hybridized C–C and sp^3^-hybridized C–C/C–H bonds. Four PMMA-related peaks can be assigned to C–H, C–C, O–CH_3_ (methoxy functional group), and O–C=O (carboxy functional group) bonds, respectively [34–36]. PMMA residues on the graphene surface mainly feature three peaks resulting from C–C bonds and carbon–oxygen-related bonds (i.e., methoxy and carboxy functional groups). After PMMA has been removed in the acetone bath, the peak of the C–H bond can rarely be observed because of a broader merger with the peak of sp^3^-hybridized C–C/C–H bonds in graphene. In Figure 4b, the peak intensities of C–C, O–CH_3_, and O–C=O bonds are, respectively, 17.6%, 14.6%, and 15.0% of the main peak intensity (sp^2^ C–C). In Figure 4c, the same ratios decrease to, respectively, 2.9%, 2.6%, and 4.8%. Therefore, regarding O–C=O bonds, the residue caused by B2 was reduced by three times and decreased about six times for the removal of C–C and O–CH_3_ bonds, implying the crucial role of PMMA-15k in the mixture.

**Figure 5:**
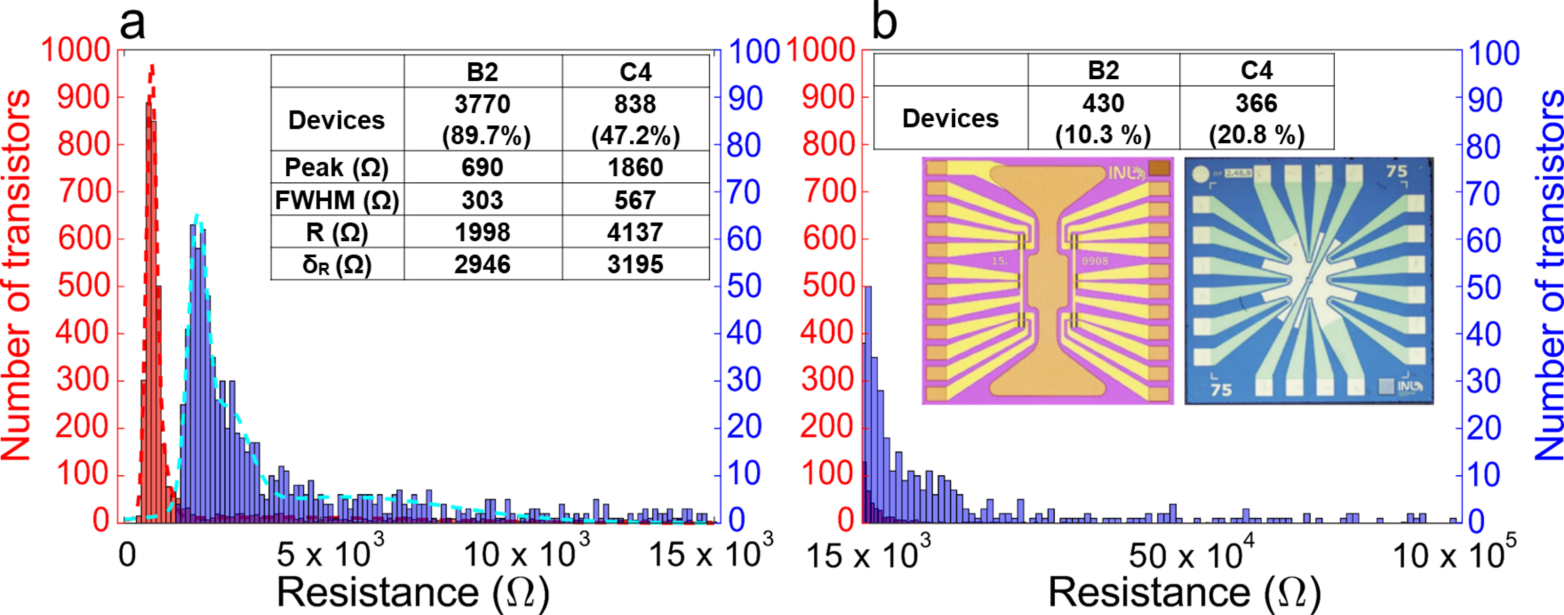
Distributions of the graphene channel resistance values in devices made using C4 (blue) and B2 (red) PMMA. The inset shows optical micrographs of representative B2 (left) and C4 (right) transistors. The graphs show the resistance distributions in (a) the 0–15 kΩ range (with 125 Ω bins) and (b) the range from 15 kΩ to 1 MΩ (10 kΩ bins). The multiple Gaussian fits identify the dominant resistance values in both batches. When considering all sub-15000 Ω transistors, the difference between the average resistances is 2139 Ω.

The optimized graphene transfer process was statistically validated in a batch fabrication context by comparing the channel resistance of electrolyte-gated graphene field-effect transistors (GFETs, Figure 5) designed to operate as DNA biosensors. Two batches of GFETs having a topmost graphene channel (75 µm width × 25 µm length) were fabricated (see details in Supporting Information File 1). In the first batch [37–38] (including 1755 GFETs), graphene was transferred with the C4 PMMA mixture (see Section 2.2). The data acquired from the first batch were used to benchmark a successive test on a more extensive second batch [39–40] (4200 GFETs) that used B2 PMMA for the graphene transfer. Figure 5 shows the resistance distribution in the two cases. The distributions were fitted with multiple Gaussian curves to identify the predominant resistance values in each transistor batch. The first Gaussian curves of C4 and B2 (which envelop the most common resistance bins, see Figure 5) peak at 1860 and 690 Ω, respectively. This is a difference of 1170 Ω. The much lower channel resistance for the B2 mixture gives further evidence of a more robust, cleaner, and more effective transfer process. The B2 transfer appears to minimize the PMMA residues, known to act as centers of carrier scattering in graphene and to increase its resistance [19–20]. The vast majority (ca. 90%) of B2 data populate the first Gaussian curve with a narrow distribution (FWHM of ca. 300 Ω). In stark contrast, the C4 data are scattered over a much broader range. Almost 50% populate a broader Gaussian curve with two peaks (FWHM of the first peak: ca. 567 Ω) in the range up to 15 kΩ, while more than 20% have values up to 1 MΩ. Differently from B2, a consistent sample subset (above 30%) is above 1 MΩ, which means that a third of the fabricated devices are open circuits due to an imperfect graphene transfer (leading to highly damaged or lacking graphene). Overall, the B2 mixture yields more consistent electric properties of the graphene channel, thanks to a homogenous and reproducible process. Such characteristics ultimately translate into a consistent sensor performance, which is pivotal for industrial fabrication.

## Conclusion

Monolayer graphene films and single crystals were transferred using PMMA with different AMWs and weight percentages in anisole. Repeated transfer cycles among water baths revealed, as expected, that PMMA with higher AMW and weight percentage allowed for a better mechanical support to graphene. Optical microscopy, Raman spectroscopy, and XPS carried out to evaluate the amount of PMMA residues on graphene after the transfer processes showed that PMMA with higher AMW resulted in a more significant number of residues: PMMA-950k AMW (C4) yielded a maximum of 180 residues (in a 650 × 500 µm^2^ area), while the optimized mixture (PMMA-15k/550k, B2) yield a minimum value of ten residues in the same area. More abundant PMMA contamination on graphene translated into a more intense p-type doping, as evidenced by (i) the position of the Raman G peak, which was blueshifted by ca. 6 cm^−1^ (between C4 and B2 samples), and (ii) the FWHM of the G peak, which appeared broader in B2 samples (23 cm^−1^ vs 14 cm^−1^ of the C4 samples). The XPS analysis showed a markedly increased presence of C–H, C–C, O–CH_3_, and O–C=O bonds in C4 graphene compared to B2 samples, corroborating the previous findings. We validated the impact of the optimized B2 process in the mass fabrication of arrays of electrolyte-gated GFETs. The channel resistances of thousands of GFETs prepared using B2 and C4 PMMA were measured with a probe station in air. The resistance distributions were analyzed and fitted with Gaussian curves. The resistance distributions were centered at 690 Ω (FWHM = 303 Ω) and 1860 Ω (FWHM = 567 Ω), respectively, proving that the optimized PMMA mixture enables the production of reproducible arrays of electronic devices with consistent properties.

## Experimental

### Graphene growth

Single-crystal and large-area graphene were obtained on Cu foil via catalyst-assisted growth in a low-pressure CVD system (CVD First Nano, EasyTube 3000). A 25 µm thick annealed Cu foil (Alfa Aesar, purity 99.8%), serving as a metal catalyst, was placed in a graphite enclosed cavity during the whole process. The temperature for annealing and growth was kept stable at 1040 °C by PID thermal controllers. The Cu foil was first annealed in argon atmosphere (500 sccm, 9.0 Torr) for 30 min in a quartz tube furnace. In the growth process, the gas mixture of argon (250 sccm), hydrogen (100 sccm), and methane (1.2 sccm) was subsequently introduced into the quartz chamber, where a reaction pressure of 4.0 Torr was kept constant through the variable frequency-driven pumping system. The growth time for single graphene crystals (250–350 µm) and large-area graphene films (ca. 25 cm^2^) was 40 and 80 min, respectively. To finalize the process, an argon flush of 500 sccm was conducted to cool down samples until the furnace reached room temperature.

### Graphene transfer

For the preparation of the various PMMA solutions, PMMA-15k (Sigma-Aldrich, 200336) and PMMA-550k (Alfa Aesar, 43982) powders were dissolved in anisole (Merck, 801452) in different proportions, as indicated in Table 1. PMMA-950k (MicroChem, PMMA-950k A4) at 4 wt % is commonly used for microfabrication processes and was chosen for comparison. The optimized PMMA mixture (coded B2) was made by mixing PMMA-550k and PMMA-15k in anisole at a 2:1 ratio (3 wt %). The solutions were stirred at 1000 rpm for 24 h. The graphene transfer process is illustrated in Supporting Information File 1, Figure S1. Graphene/Cu foil was spin-coated with PMMA at 3000 rpm for 30 s, followed by drying in a fume hood for 8 h. The back side graphene was removed by oxygen plasma (4 × 10^−1^ mbar, 250 W, 90 s).

**Table 1:** PMMA solutions used in the graphene transfer tests.

Name	Ratio (wt %)	Polymer AMW	Polymer wt %	Anisole mass (g)

A1	2	15,000	2	98
C1	4	15,000	4	96
A3	2	550,000	2	98
C3	4	550,000	4	96
C4	4	950,000	4	96
B2	3	15,000/550,000	1/2	97

PMMA/graphene/Cu foil was placed over the 0.5 M FeCl_3_ solution surface for 3 h to etch away the Cu foil. After the etching process, PMMA/graphene floating on the surface of the FeCl_3_ solution was rinsed with DI water three times. A rinse in 2% HCl solution was done to remove metal precipitates. At last, the PMMA/graphene was washed in DI water three times and scooped up with a target substrate (SiO_2_/Si wafer). The sample was dried in a vacuum chamber (ca. 10^−4^ Torr) at room temperature for 2 h. For PMMA removal, the entire sample was vertically dipped into an acetone bath for 4 h. After that, the exposed graphene on a SiO_2_/Si substrate was again vertically dipped into IPA and then DI water bath for 1 h. Finally, the graphene on the receiving substrate was blow-dried with N_2_.

### Optical microscopy

A selective oxidation method was adopted to rapidly identify the as-grown graphene, enabling the direct optical inspection of the graphene domains without the laborious transfer process. Following this method, the Cu substrate with graphene was first oxidized in ambient air on a hot plate at 200 °C for 2 min. The graphene film on the Cu substrate serves as a protection layer, preventing the underlying Cu surface from oxidation because of its high chemical/thermal stability and impermeability to gases and liquids. In contrast, the surrounding exposed areas of the Cu foil surface exhibited high reactivity and were readily oxidized to copper oxides with a noticeable color change. The apparent color contrast between the oxidized and non-oxidized Cu surfaces made the synthesized graphene domains easy to be observed in an optical microscope equipped with a CCD camera (Supporting Information File 1, Figure S1a).

### Raman spectroscopy

Large-area graphene films and single graphene crystals transferred onto SiO_2_/Si substrates were characterized by Raman microscopy (WITec GmbH, Model: Alpha300M+) with an excitation laser wavelength of 532 nm. A laser power of ca. 2 mW was used for all measurements. The backscattered laser light containing the Raman bands from 1200 to 3000 cm^−1^ was collected by a CCD camera (Andor, Model number: DV401A-BV-352) integrated with the WITec system. The characteristic Raman signature collected from a p-doped Si wafer at 520.7 cm^−1^ was employed as standard calibration. Raman mapping was conducted by raster scan, where the step size of the laser spot moving over a selected area is 1 µm, and the exposure time of 0.4 s was taken at each point of the mapping. In the maps, the intensity of D and 2D bands were normalized to the G band intensity. Corresponding statistics were extracted from the maps.

### X-ray photoelectron spectroscopy

The chemical components of PMMA residues were analyzed by X-ray photoelectron spectroscopy (XPS, Thermo Scientific ESCALAB 250Xi) using a non-monochromatic Mg Kα source with an analysis spot smaller than 2 mm^2^. The detection system contains a double-focusing 180° spherical sector analyzer with a mean radius of 150 mm and an energy range of 0 to 5 keV. The pressure in the analysis chamber was ca. 5 × 10^−10^ Torr, and the analyzer had a pass energy of 20 eV.

### Atomic force microscopy

The surface topographies of graphene were investigated by a Bruker Dimension Icon atomic force microscope (AFM), using PPP-NCH (NanosensorsTM) cantilevers with a tip radius smaller than 20 nm, a force constant of 42 N/m, and 250 kHz resonance frequency. The AFM measurement was carried out in tapping mode. A 633 nm laser light aimed at the back side of the cantilever tip was reflected toward a position-sensitive photodetector, which provides feedback signals to piezoelectric scanners that maintain the cantilever tip at constant height (force) above the surface, thus, reproducing its topography.

### Fabrication and characterization of graphene field-effect transistors

Receded-gate graphene field-effect transistors were fabricated on an 8″ Si/SiO_2_ (200 nm thick) wafers. Two arrays of devices were fabricated with different process steps (Wafer 1: C4 PMMA; Wafer 2: B2 PMMA). Both wafers started with the patterning of Cr/Au contacts (deposited by magnetron sputtering) using direct-write laser lithography and ion milling. The fabrication of the two wafers followed slightly different steps, as described below.

**Wafer 1:** A stopping layer (Al_2_O_3_/TiWN/AlSiCu/TiWN) was patterned by lift-off, followed by the CVD growth of a multi-stack layer of SiO_2_ and Si_3_N_4_ to passivate the current lines. After this, a thin Al_2_O_3_ layer was deposited by sputtering and patterned by wet etching to protect the gate during the graphene etch. The C4 PMMA/graphene films were then transferred onto the patterned wafer until all device areas were covered. After removing the PMMA, graphene was patterned using optical lithography and oxygen plasma etching. Finally, the sacrificial layer was removed by wet etching.

**Wafer 2:** An additional layer of Al_2_O_3_ was deposited on the Au layer as protection. After that, the two wafers followed different fabrication processes. A residue-free transfer process was used, using a sacrificial metallic mask (TiWN, AlSiCu, TiWN) patterned by lift-off to protect the entire wafer except for the areas around the channel, source, and drain, on which the graphene film would make electrical contact. The B2 PMMA/graphene films were then transferred onto the wafer and patterned by O_2_ plasma, followed by the sacrificial layer removal. Previous to the passivation, Al_2_O_3_ was selectively removed to improve the adhesion of the oxide passivation to the surface of the chips. A stopping layer (Cu/AlSiCu/TiW) for the reactive ion etching (RIE) process was sputtered, and the SiO_2_/SiN*_x_* multistack passivation layer was deposited by CVD. The passivation layer was patterned by lithography and etched by RIE until revealing the stopping layer on the contact pads and graphene transistor channels. Finally, the stopping layer was removed by wet etching, exposing the graphene channel.

In both wafers, the GFET channels had nominal dimensions of *W* = 75 µm and *L* = 25 µm. The total number of GFETs condidered in this study was 1755 on Wafer 1 and 4200 on Wafer 2. The source–drain resistance of the GFETs was measured with a semi-automatic DC measurement probe station for 8″ wafers. The setup uses a 40-tip probe head with 250 µm spacing between the tips; each probe allowed us to measure 20 devices simultaneously. A current was injected into each transistor to reach an output voltage of 1 mV. Histogram distributions for the graphene channel resistances were plotted (using MATLAB scripts) to compare the effects of B2 and C4 PMMA-assisted graphene transfer on device performance. Based on our observations of the device performance, a threshold resistance of 15000 Ω was selected as the cut-off resistance value identifying working graphene channels (i.e., correctly operating devices). A maximum resistance value of 1 MΩ was set to identify the non-operating channels (due to imperfect transfer leading to heavily damaged or lacking graphene). Multiple Gaussian fits were performed to isolate the trends in the resistance distributions.

## Supporting Information

File 1 Additional experimental data.

## References

[1] Butler S Z, Hollen S M, Cao L, Cui Y, Gupta J A, Gutiérrez H R, Heinz T F, Hong S S, Huang J, Ismach A F (2013). ACS Nano.

[2] Geim A K, Grigorieva I V (2013). Nature.

[3] Novoselov K S, Mishchenko A, Carvalho A, Castro Neto A H (2016). Science.

[4] Obraztsov A N (2009). Nat Nanotechnol.

[5] Hao Y, Bharathi M S, Wang L, Liu Y, Chen H, Nie S, Wang X, Chou H, Tan C, Fallahazad B (2013). Science.

[6] Chen C-C, Kuo C-J, Liao C-D, Chang C-F, Tseng C-A, Liu C-R, Chen Y-T (2015). Chem Mater.

[7] Han T-H, Lee Y, Choi M-R, Woo S-H, Bae S-H, Hong B H, Ahn J-H, Lee T-W (2012). Nat Photonics.

[8] Britnell L, Ribeiro R M, Eckmann A, Jalil R, Belle B D, Mishchenko A, Kim Y-J, Gorbachev R V, Georgiou T, Morozov S V (2013). Science.

[9] Gomez De Arco L, Zhang Y, Schlenker C W, Ryu K, Thompson M E, Zhou C (2010). ACS Nano.

[10] Chen M, Haddon R C, Yan R, Bekyarova E (2017). Mater Horiz.

[11] Liang X, Sperling B A, Calizo I, Cheng G, Hacker C A, Zhang Q, Obeng Y, Yan K, Peng H, Li Q (2011). ACS Nano.

[12] Kim S M, Hsu A, Lee Y-H, Dresselhaus M, Palacios T, Kim K K, Kong J (2013). Nanotechnology.

[13] Kim S, Shin S, Kim T, Du H, Song M, Lee C, Kim K, Cho S, Seo D H, Seo S (2016). Carbon.

[14] Lin Y-C, Lu C-C, Yeh C-H, Jin C, Suenaga K, Chiu P-W (2012). Nano Lett.

[15] Borin Barin G, Song Y, de Fátima Gimenez I, Souza Filho A G, Barreto L S, Kong J (2015). Carbon.

[16] Capasso A, De Francesco M, Leoni E, Dikonimos T, Buonocore F, Lancellotti L, Bobeico E, Sarto M S, Tamburrano A, De Bellis G (2014). Appl Phys Lett.

[17] Ullah S, Yang X, Ta H Q, Hasan M, Bachmatiuk A, Tokarska K, Trzebicka B, Fu L, Rummeli M H (2021). Nano Res.

[18] Qing F, Zhang Y, Niu Y, Stehle R, Chen Y, Li X (2020). Nanoscale.

[19] Suk J W, Lee W H, Lee J, Chou H, Piner R D, Hao Y, Akinwande D, Ruoff R S (2013). Nano Lett.

[20] Pirkle A, Chan J, Venugopal A, Hinojos D, Magnuson C W, McDonnell S, Colombo L, Vogel E M, Ruoff R S, Wallace R M (2011). Appl Phys Lett.

[21] Ahn Y, Kim H, Kim Y-H, Yi Y, Kim S-I (2013). Appl Phys Lett.

[22] Jeong H J, Kim H Y, Jeong S Y, Han J T, Baeg K-J, Hwang J Y, Lee G-W (2014). Carbon.

[23] Malard L M, Pimenta M A, Dresselhaus G, Dresselhaus M S (2009). Phys Rep.

[24] Ferrari A C, Basko D M (2013). Nat Nanotechnol.

[25] Pimenta M A, Dresselhaus G, Dresselhaus M S, Cançado L G, Jorio A, Saito R (2007). Phys Chem Chem Phys.

[26] Faggio G, Capasso A, Messina G, Santangelo S, Dikonimos T, Gagliardi S, Giorgi R, Morandi V, Ortolani L, Lisi N (2013). J Phys Chem C.

[27] Lisi N, Buonocore F, Dikonimos T, Leoni E, Faggio G, Messina G, Morandi V, Ortolani L, Capasso A (2014). Thin Solid Films.

[28] Gnisci A, Faggio G, Messina G, Kwon J, Lee J-Y, Lee G-H, Dikonimos T, Lisi N, Capasso A (2018). J Phys Chem C.

[29] Ni Z H, Wang H M, Luo Z Q, Wang Y Y, Yu T, Wu Y H, Shen Z X (2010). J Raman Spectrosc.

[30] Das A, Pisana S, Chakraborty B, Piscanec S, Saha S K, Waghmare U V, Novoselov K S, Krishnamurthy H R, Geim A K, Ferrari A C (2008). Nat Nanotechnol.

[31] Wu J-B, Lin M-L, Cong X, Liu H-N, Tan P-H (2018). Chem Soc Rev.

[32] Zhuang B, Li S, Li S, Yin J (2021). Carbon.

[33] Capasso A, Dikonimos T, Sarto F, Tamburrano A, De Bellis G, Sarto M S, Faggio G, Malara A, Messina G, Lisi N (2015). Beilstein J Nanotechnol.

[34] Ton-That C, Shard A G, Teare D O H, Bradley R H (2001). Polymer.

[35] Ferrah D, Renault O, Petit-Etienne C, Okuno H, Berne C, Bouchiat V, Cunge G (2016). Surf Interface Anal.

[36] Cunge G, Ferrah D, Petit-Etienne C, Davydova A, Okuno H, Kalita D, Bouchiat V, Renault O (2015). J Appl Phys.

[37] Campos R, Borme J, Guerreiro J R, Machado G, Cerqueira M F, Petrovykh D Y, Alpuim P (2019). ACS Sens.

[38] Motoso Abreu C F (2019). Biosensors for Enhanced in Vitro Fertilisation Outcomes.

[39] Cabral P D, Domingues T, Machado G, Chicharo A, Cerqueira F, Fernandes E, Athayde E, Alpuim P, Borme J (2020). Materials.

[40] Purwidyantri A, Domingues T, Borme J, Guerreiro J R, Ipatov A, Abreu C M, Martins M, Alpuim P, Prado M (2021). Biosensors.

