# Supplementary material for: Optimizing PMMA solutions to suppress contamination in the transfer of CVD graphene for batch production
Source: Beilstein J Nanotechnol. 2022 Aug 18;13:796–806. doi: 10.3762/bjnano.13.70 (PMC9443383; doi:10.3762/bjnano.13.70)
Supplement: File 1 — Additional experimental data. [file Beilstein_J_Nanotechnol-13-796-s001.pdf]

BEILSTEIN JOURNAL OF NANOTECHNOLOGY

Supporting Information

for

Optimizing PMMA solutions to suppress contamination in the transfer of CVD graphene for batch production

Chun-Da Liao, Andrea Capasso, Tiago Queirós, Telma Domingues, Fatima Cerqueira, Nicoleta Nicoara, Jérôme Borme, Paulo Freitas and Pedro Alpuim

Beilstein J. Nanotechnol. **2022,** *13,* 796–806. doi:10.3762/bjnano.13.70

Additional experimental data

License and Terms: This is a supporting information file under the terms of the Creative Commons Attribution License (https://creativecommons.org/licenses/by/4.0). Please note that the reuse, redistribution and reproduction in particular requires that the author(s) and source are credited and that individual graphics may be subject to special legal provisions.
The license is subject to the *Beilstein Journal of Nanotechnology* terms and conditions: (https://www.beilstein-journals.org/bjnano/terms)

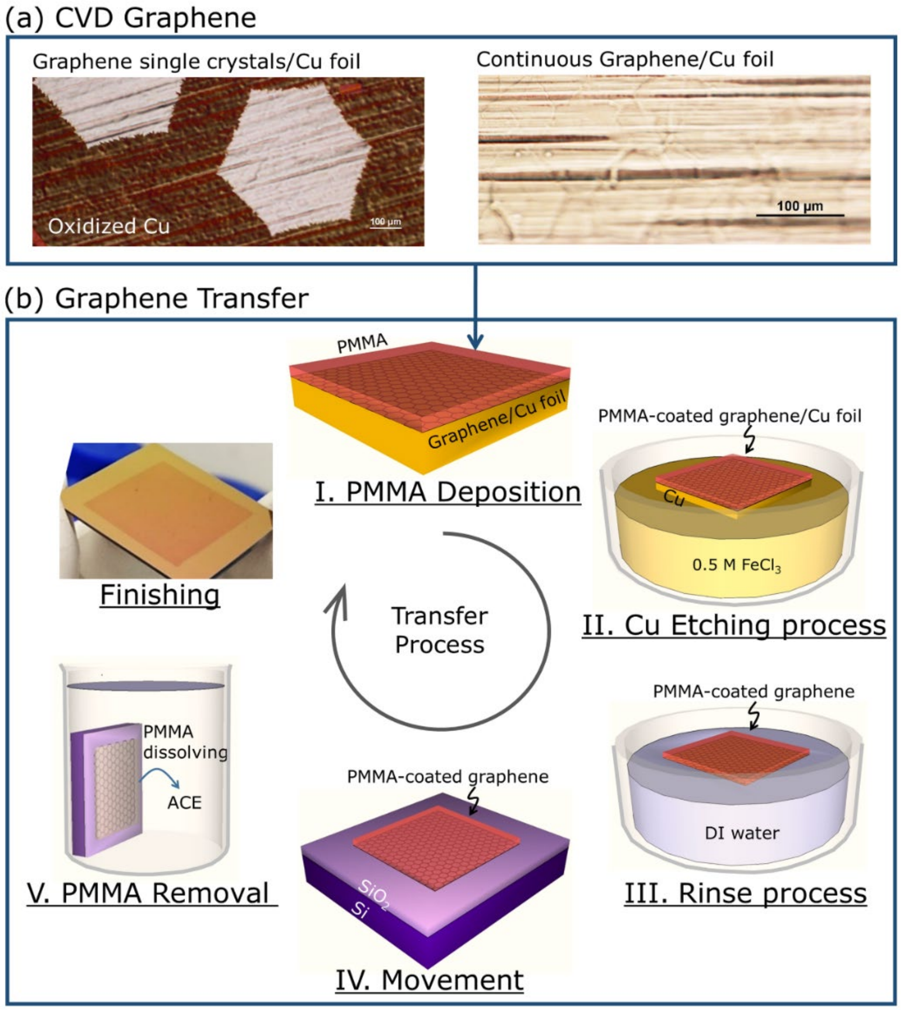

Figure S1: Transfer of CVD graphene. (a) The Cu foils serving as metal catalysts are used to grow single graphene crystals and large-area graphene films. (b) A conventional process of graphene transfer is illustrated. In step (I) the PMMA supporting layer is spin-coated on the graphene surface; in step (II), the Cu substrate is subsequently etched away in 0.5 M $FeCl_3$ solution. In step (III), PMMA-coated graphene is rinsed in DI water bath. After the rinse process, displayed in step (IV), PMMA-coated graphene is scooped up and laid on the surface of the target substrate. In step (V), the removal process of PMMA supporting layer is conducted in an acetone bath.

After single graphene crystals were grown on Cu foils through CVD, a selected oxidation process was conducted to examine the size of the as-grown graphene. As shown in Figure S1a, the average lateral size of single graphene crystals used for the transfer process is around 250–350 μm in this study. A conventional graphene wet transfer process (Figure S1b) involves (I) the deposition of PMMA supporting layers, (II) the

etching of metallic substrates, (III) the rinse of PMMA-coated graphene, (IV) the movement of PMMA-coated graphene lying onto the surface of the target substrate, and (V) the removal of PMMA supporting layers. In step (I), conventional heat treatment on PMMA/graphene/Cu foils is conducted. Either soft (80–90 °C) or hard bake (120–180 °C) is used to intensify the polymerization of the as-coated PMMA supporting layer. Also, in step (IV), the baking treatment is commonly executed to increase the contact and expel the extra water between graphene and the target substrate. PMMA is heated accordingly, increasing the residue remaining on the graphene surface. Therefore, the first claim in our improved transfer process is the absence of thermal treatment. The spin-coated PMMA on graphene was dried in a chemical fume hood for 6 h instead of the baking process. In addition, the SiO_2/Si substrate carrying a piece of PMMA-coated graphene leaned against the beaker at an angle of over 60 °C. The water between the as-transferred graphene and the SiO_2/Si substrate slowly leaked out from the bottom edge of the sample. After the PMMA-coated graphene was dried on SiO_2/Si substrate, the entire sample was moved into a vacuum chamber and evacuated to a pressure of 1×10^{-4} Torr for 2 h. The purpose is to eliminate extra water and increase contact without additional thermal treatment.

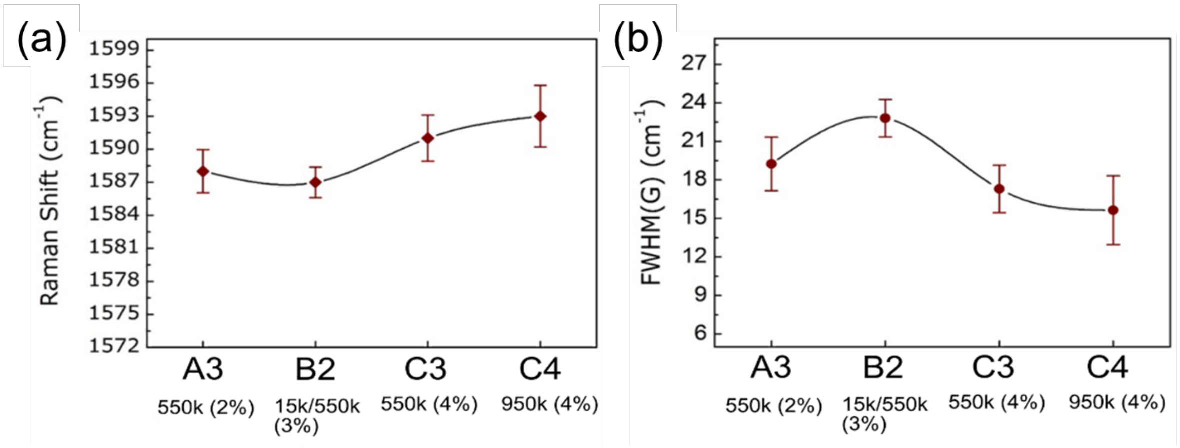

Figure S2: (a) Average G mode Raman shift peak position for different PMMA mixtures. (b) Average FWHM of G mode Raman shift peak for different PMMA mixtures.

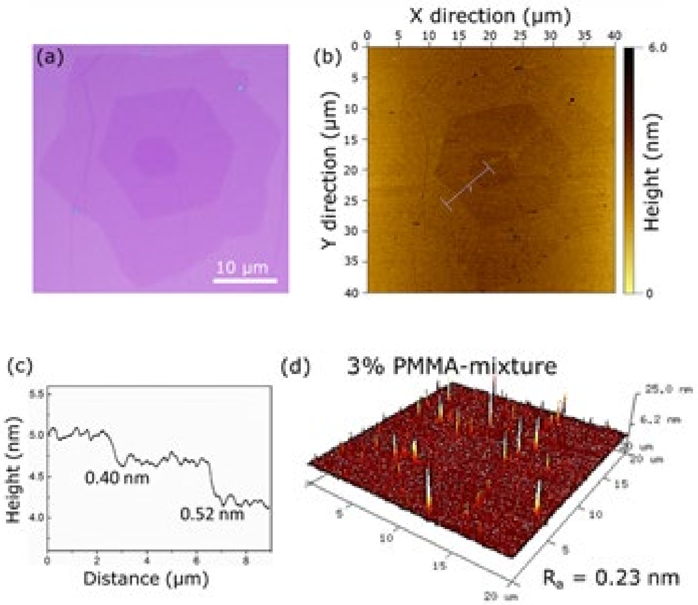

Figure S3: Graphene crystal transferred with B2 PMMA. (a) Optical and (b) AFM image of the crystal. (c) Height profile along the white line in (b). (d) 3D surface topography (20 × 20 μm^2) and average surface roughness analysis.

The optical image in Figure S3a shows a graphene crystal transferred with B2 PMMA. The crystal is made of four layers. The AFM image (Figure S3b) highlights the three topmost layers, almost homocentric and with a hexagonal shape. The thickness of the two topmost layers is 0.4 and 0.5 nm (Figure S3c). The morphology of the crystal appears very smooth and free of identifiable impurities. The average surface roughness (R_a) of a 20 × 20 μm^2 area of the transferred crystals is ca. 0.2 nm (Figure S3d), that is, much lower than the average R_a of crystals transferred with C4 PMMA (ca. 2.8 nm).
